# Role of Adverse Childhood Experiences and Intersecting Identities on Adolescents' School Engagement in the United States*

**DOI:** 10.1111/josh.70072

**Published:** 2025-08-20

**Authors:** Juhee K. Cavins, Hye Yeon Lee, Isak Kim

**Affiliations:** ^1^ Department of Educational Psychology and Learning Systems Florida State University Tallahassee Florida USA; ^2^ Center for Teaching and Learning Georgia Institute of Technology Atlanta Georgia USA; ^3^ Department of Counselor Education University of Iowa Iowa City Iowa USA

**Keywords:** adverse childhood experiences, intersecting identities, school engagement

## Abstract

**Background:**

The purpose of this study was to investigate the role of adverse childhood experiences (i.e., ACEs) and intersecting identities (i.e., gender and ethnicity) on school engagement among adolescents in the United States.

**Methods:**

We analyzed the 2021 National Survey of Children's Health Data.

**Results:**

We first identified four ACE classes, with each class representing different proportions of intersecting identities: *No ACEs, Multiple Low Risk, Mental Health Issues, and Multiple High Risk*. We then found significant differences in school engagement across the ACE class memberships and eight different intersecting identities, both separately and together. When we investigated ACE class memberships and intersecting identities separately, the results underscored the pervasive negative impact of the *Multiple High Risk* class on school engagement across all intersecting identities. Regarding intersecting identities, Asian female adolescents exhibited the highest school engagement levels, while White male adolescents had the lowest. When considering both ACE class memberships and intersecting identities, Asian male, Black female, Black male, Hispanic male, and White male adolescents in the *Multiple High Risk* class demonstrated lower school engagement levels, which contrasted with the results when examining only either intersecting identities or ACEs.

**Implications for School Health Policy, Practice, and Equity:**

These findings highlight the importance of addressing both factors in school health policies and practices to better support various adolescent populations.

**Conclusions:**

Our study provides a foundation for future studies and informs the development of more equitable, trauma‐informed school policies and practices to foster student engagement.

## 1 Introduction

Active school engagement, such as regular attendance, active class participation, and completion of assignments, plays a key indicator of academic success [1, 2, 3]. High levels of school engagement are often correlated with improved academic performance [1, 3]. Low levels of engagement are associated with underachievement, behavioral challenges, and a heightened risk of dropping out [2, 4]. Despite the importance of school engagement, there is a growing trend of disengagement from school activities (e.g., showing boredom, zoning out, sleeping, and using digital devices during class [5, 6, 7, 8, 9]), particularly as students progress from elementary to middle to high school [10, 11]. Due to these escalating concerns, researchers have delved into various factors influencing K‐12 students' school engagement. For example, some researchers have focused on contextual factors (e.g., Adverse Childhood Experiences or ACEs [12]), while others have delved into the individual factors (e.g., intersecting identities, including gender and ethnicity [13]). Although each factor provides unique and complementary insights into the dynamics influencing adolescents' school engagement [14], none of the studies have examined the combined impacts of contextual and individual factors on school engagement, leading to a lack of nuanced understanding. To address this gap, we investigated the role of ACEs (e.g., domestic violence, drug use in the household) and intersecting identities, including gender and ethnicity (e.g., Asian female, Hispanic male), both together and seperately, on school engagement. We believe that this approach offers a more comprehensitve understanding of adolescents' school engagement, in particular for those who may need more targeted and supportive educational interventions.

### 1.1 School Engagement

School engagement, viewed as vital for students' success in school, has been defined and measured in various ways as an attempt to examine students' involvement and commitment to school [15]. One definition describes school engagement as a multifaceted concept encompassing behavioral, emotional, cognitive, and/or motivational dimensions [16, 17]. The behavioral component includes commitment, attendance, and participation in class, whereas the emotional dimension pertains to students' feelings toward their peers, teachers, and schools. The cognitive aspect refers to students' thoughts and beliefs about school and their abilities, and the motivational aspect reflects a student's drive to succeed [15].

Our study particularly focused on the behavioral component of school engagement due to the structure of our secondary data and the potential overlap among the dimensions [16, 18]. Specifically, the secondary data included commonly used observable indicators, such as commitment to success (e.g., what are the levels of commitment to school or success?) and academic participation (e.g., completing homework [17]). Further, the potential overlap among the dimensions may lead to the use of numerous similar but slightly different definitions and measurements, contributing to conceptual ambiguity [16]. Therefore, we provided a more straightforward assessment of school engagement by focusing on the behavioral component while complementing our analysis with contextual (e.g., ACEs) and individual factors (e.g., intersecting identities) closely linked to varying levels of school engagement.

### 1.2 Adverse Childhood Experiences and School Engagement

Within the realm of contextual factors, scholars have examined the association of school engagement with childhood adversity, trauma, and experiences in dysfunctional families as risk factors (i.e., Adverse Childhood Experiences or ACEs [19]). ACEs refer to potentially traumatic events occurring in childhood before the age of 18 [19]. ACEs encompass a range of childhood events, including but not limited to child maltreatment (e.g., abuse, neglect), various forms of household dysfunction (e.g., income hardship, parental death, parental divorce/separation, family member's incarceration), and adversities within the school and community environments (e.g., exposure to violence and discrimination [18, 19, 20]). Traditional ACEs assessment have assessed individuals' traumatic events, such as maltreatment in children (e.g., physical abuse) and household dysfunction (e.g., living with parents who have a mental illness or are addicted to substance abuse). However, other components of ACEs (e.g., gender and race/ethnicity) have been also considered because discriminatory experiences associated with these factors have been detrimentally associated with adolescents' overall development, including their health and well‐being [21, 22]. This necessitates the inclusion of such additional items in the ACEs.

Exposure to ACEs reflects distinctive familial and contextual environments that shape adolescents' active school engagement. Distinctive exposure to more than one ACEs is considered as representing varying ecological conditions [14], affecting adolescents' ability and willingness to participate actively in school activities. Indeed, prior research has documented that ACEs can adversely affect school engagement and serve as challenges for school success, demonstrating the dose–response relationship (i.e., the relationship between greater numbers of cumulative ACEs and increased risks of disengagement in school) [12, 23, 24]. In addition to the dose–response relationship, certain types of ACEs can be more detrimental to students than others. For instance, prior research [25] has established that household income hardship and parental divorce/separation were more significantly associated with lower school engagement than other types of ACEs. These results underscore the importance of further investigating the association between heterogeneous patterns of ACEs and school engagement. To address this gap, we aimed to identify underlying patterns of ACEs and their associations with school engagement, while also considering individual factors, such as intersecting identities.

### 1.3 Intersecting Identities and School Engagement

Among individual factors associated with school engagement, we focused on adolescents' intersecting identities, specifically gender and race/ethnicity [26, 27]. This framework proposes that an individual's social identities, such as gender and race/ethnicity, are deeply interconnected with one another, rather than functioning in isolation [27, 28]. In other words, the dynamics shaped by both gender and race/ethnicity are considered more complicated than those shaped by gender or race/ethnicity separately [27, 28]. This concept of intersecting identities has also been used in educational settings to better understand how school experiences vary among students from various backgrounds [29, 30, 31]. Specifically, students' intersecting identities have been linked to varying levels of school engagement. For instance, previous studies on gender differences have consistently shown that female adolescents tend to exhibit higher levels of school engagement compared to their male counterparts [4, 32]. These results may be due to female adolescents' greater academic motivation, leading to increased academic effort in school [33, 34]. Research on ethnicity, on the other hand, has yielded mixed results. For instance, one study [35] found that Black adolescents showed higher levels of school engagement compared to other ethnic groups, such as White or Hispanic adolescents, while another study [36] found higher mean scores of school engagement among Asian and White adolescents and lower mean scores among Black and Hispanic peers.

These varied results underscore the importance of students' intersecting identities in examining their school engagement [5, 35], in addition to ACEs. Although intersecting identities and ACEs have been explored together in other fields (e.g., public health) [37, 38], there is a lack of such research in the context of education (cf. examining students' ACEs and school‐based interventions [39, 40]). Specifically, research in education has often focused on adolescents with certain intersecting identities from middle‐class families who experience minimal adversity, excluding those facing difficulties [4, 32, 41]. To fill this gap, our investigation delved into the impact of adolescents' intersecting identities on school engagement through the lens of ACEs.

## 2 Present Study

We aimed to offer a more nuanced perspective on understanding adolescents' school engagement by considering the contexual factors (i.e., ACEs) and individual factors (i.e., intersecting identities) both together and separately. To achieve this goal, we propose five research questions:

1. What are the latent classes of adolescents aged 12–17 years, including both with and without ACEs?
2. Do the levels of adolescents' school engagement differ across their ACE class memberships?
3. Do the levels of adolescents' school engagement differ across intersecting identities by gender and race/ethnicity?
4. What are the associations between adolescents' ACE class memberships and intersecting identities?
5. What are the associations of adolescents' ACE class memberships and intersecting identities in terms of levels of school engagement?

## 3 Method

### 3.1 Survey Data and Sample

The current investigation used large cross‐sectional secondary data from the 2021 National Survey of Children's Health (NSCH) [18]. The NSCH survey data were sponsored and directed by the Health Resources and Service Administrator's Maternal and Child Health Bureau (HRSA MCHB). The respondents of the 2021 NSCH data were any parents or caregivers who live with one or more children aged 0–18 years old across the United States. The NSCH data were designed to represent the estimates of the population of the United States, including each of the 50 states and the District of Columbia. The 2021 NSCH data collection approach consisted of two phases: (a) an initial screening to assess children's basic demographic characteristics (e.g., whether one or more children aged 0–17 years lived in the household) and special health care needs and (b) topical questionnaires addressing children's physical and mental health, health insurance coverage, health care access and quality, community and family health activities, and neighborhood support, focusing on one child from each household. The NSCH 2021 was completed online or by mail between June 25, 2021 and January 14, 2022. The overall response rate for the survey was 40.3%. A higher proportion of respondents completed the survey online (90.6%), while 9.4% of the total sample completed the survey using a paper instrument. The survey data were weighted to reflect the demographic composition of children aged 0–17 years in the District of Columbia and each state, clustering, and sampling strata, which were included in the analyses. Our sample for this study was 14,946 (92.5%) out of 16,166 adolescents aged 12–17 years old after removing respondents who did not report their children's race/ethnicity. We did not seek IRB approval because this study used secondary data collected by the NSCH, which already directly obtained ethical approval and informed consent from study respondents.

### 3.2 Measures

#### 3.2.1 Adverse Childhood Experiences (ACEs)

In the 2021 NSCH data, parents or caregivers of adolescents aged 12–17 years old were asked to report about their children's exposure to 11 ACEs since their children were born: (1) household income hardship, (2) parent or guardian's divorce (or separate household), (3) parent or guardian's death, (4) parent or guardian served time in jail, (5) adolescents witnessing or hearing parent or guardian's aggressive behaviors, (6) adolescents witnessing or violence, or were victims of violence in the neighborhood, (7) adolescents living with anyone who was mentally ill, suicidal, or severely depressed, (8) adolescents living with anyone who had a problem with alcohol or drugs, adolescents treated unfairly due to (9) their race/ethnicity, (10) their sexual orientation or gender identity, and (11) their health status or disability. The ACEs questionnaires were all dichotomous (i.e., Yes, No), except for household income hardship (i.e., Very Often, Somewhat Often, Rarely, Never). To maintain consistency with other ACEs questionnaires, we re‐coded the household income hardship responses into a binary format (i.e., Yes for Very Often or Somewhat Often, No for Rarely or Never). As such, 11 dichotomous ACE questionnaires were used for analysis.

#### 3.2.2 Intersecting Identities

We focused on the intersection between gender (i.e., female, male) and four racial/ethnic groups (i.e., Asian, Black, Hispanic, White). While the NSCH 2021 data collected and categorized respondents into seven races/ethnicities, we excluded three races/ethnicities in the analysis to account for the low proportion of respondent rates (e.g., American Indian or Alaska Native, *n* = 126, 0.8%; Native Hawaiian and Other Pacific Islander, *n* = 52, 0.3%) and to proceed with comparisons among single‐race groups (two or more races, *n* = 1042, 6.4%). Thus, we analyzed eight intersecting identities in this study: (1) Asian female, (2) Asian male, (3) Black female, (4) Black male, (5) Hispanic female, (6) Hispanic male, (7) White female, and (8) White male.

#### 3.2.3 School Engagement

School engagement was assessed using two items: (a) adolescents' levels of commitment to academic success and (b) adolescents' levels of completion of required homework. Respondents rated each item on a four‐point scale, ranging from 1 (Never) to 4 (Always). The average scores for these two items were 3.28 (SD = 0.77) out of 4. The skewness value was −0.87, and the kurtosis value was −0.06. Cronbach's *α* reliability for these two items was 0.86.

### 3.3 Statistical Analyses

In the current investigation, we conducted inferential statistical analyses in multiple ways, in response to each research question. First (RQ1), we employed a person‐centered approach (i.e., Latent Class Analysis, LCA, in this study), rather than a traditional, variable‐centered, and dose‐response approach. While the variable‐centered approach has been commonly used to examine the association between the cumulative number of ACEs and other variables of interest [19], this approach may overlook the potential heterogeneity across specific patterns of ACEs experienced by adolescents. LCA addresses this limitation by identifying distinct groups of subpopulations that share similar characteristics of indicators (i.e., ACEs in this study [42]). Identifying this heterogeneity is essential because adolescents may experience different ACE expoure that cannot be adequately understood by simply counting the cumulative number of ACEs. This approach was also commonly used in prior work to profile adolescents' heterogeneous combinations of ACEs [43, 44]. We ran a series of LCAs from 2‐class through 4‐class for adolescents, adopting sampling weight, strata, and clustering, using Mplus 8.4 [45]. To determine the best class membership, we used the following indicators: (a) a lower Bayesian Information Criterion (BIC) value, (b) an entropy value close to 1, (c) a significant *p*‐value of Vuong–Lo–Mendell–Rubin likelihood ratio test (VLMR) [46], and (d) the classification ratio of each class, ensuring that each class comprised at least 5% of the total sample [47]. Second (RQ2), we ran a three‐step BCH approach in latent class analyses to investigate differences in school engagement across the four latent ACE classes. Third (RQ3), we ran Wald Chi‐square tests to examine differences in school engagement across eight predefined groups (i.e., eight intersecting identities). Fourth (RQ4), we conducted Chi‐squared tests to examine the associations between adolescents' ACE class memberships and their intersecting identities. Lastly (RQ5), we ran a hierarchical regression analysis to examine the association between ACE class membership, intersecting identities, and school engagement. All analyses accounted for the complex survey design, including sampling weights, stratification, and clustering. This is because we aimed to examine the U.S. population, rather than examining the associations among variables of interest.

## 4 Results

### 4.1 RQ1: ACE Class Memberships

**FIGURE 1:**
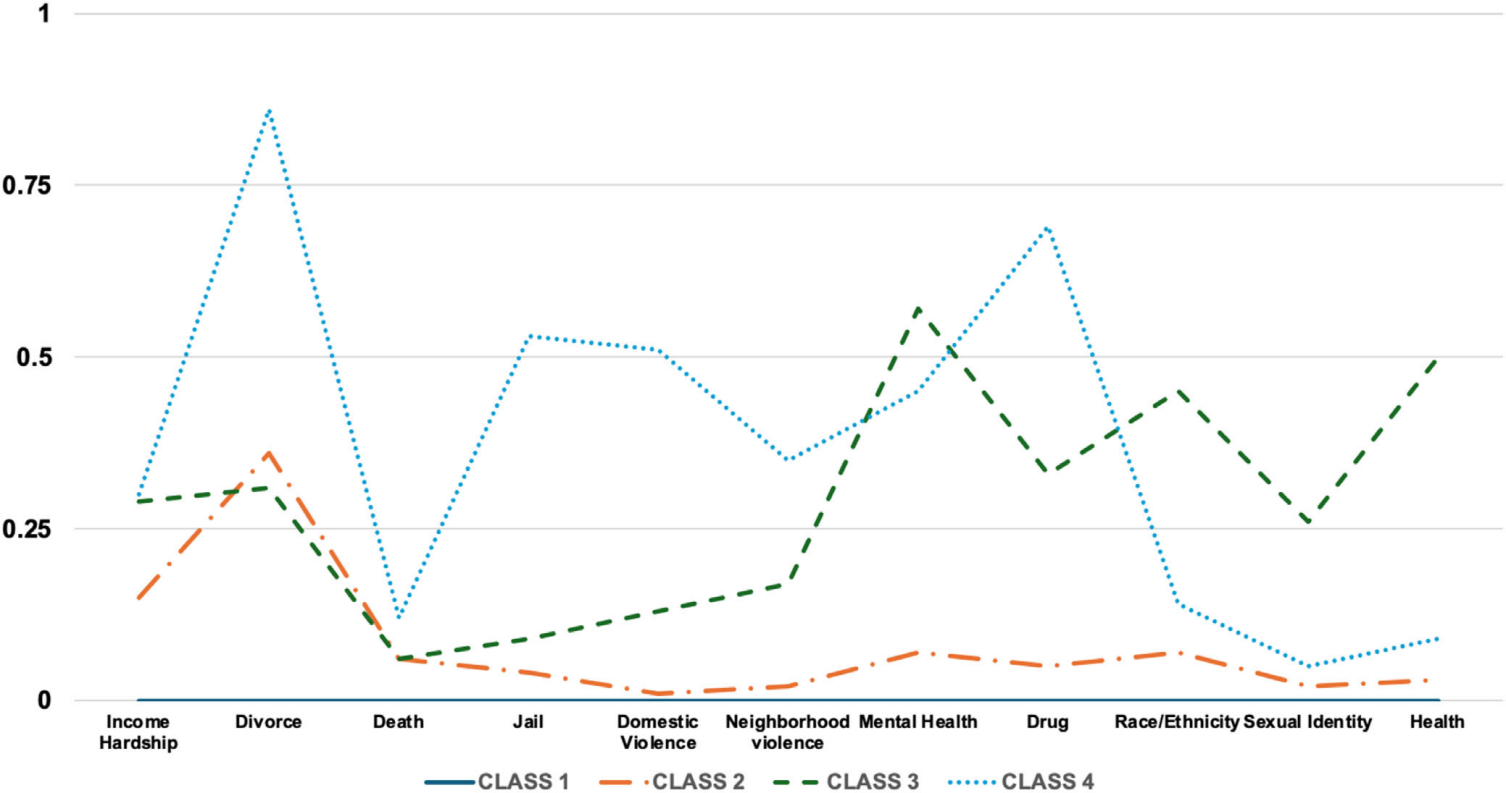
Latent class memberships of adverse childhood experiences. *Note*: Class 1: No ACEs; Class 2: Multiple Low Risk; Class 3: Mental Health Issues; Class 4: Multiple High Risk.

We conducted a series of latent class analyses from 2‐class through 4‐class to identify class memberships for adolescents aged 12–17 years, including both with and without ACEs. To determine the best model fit, we employed a comprehensive evaluation process, considering various criteria, such as BIC, Entropy, *p*‐values of LMR (VLMR), and the 5% rule of class ratio (see Table 1). Overall, the BIC value was likely to decrease once the number of latent groups increased. That is, the 4‐class model had a lower BIC value compared to the 3‐class model. Second, the 4‐class model demonstrated higher entropy compared to the 3‐class model. Third, the *p*‐values of the LMR/VLMR tests for both the 3‐class and 4‐class models were non‐significant. Fourth, the 2‐class and 3‐class models satisfied the 5% rule; the 4‐class did not. Based on these results, we proceeded with the 4‐class model based on the following considerations, even with the violation of the 5% rule. This is because small classes are not necessarily problematic in large samples if they are well‐separated and theoretically meaningful, even if they are composed of less than 5% of the sample [42, 48]. As shown in Figure 1, Class 1 was termed *No ACEs* (26.9%) and included individuals with no ACEs experience. Class 2 was named *Multiple Low Risk* class (58.7%) and included multiple ACEs but overall low risk. Class 3, named *Mental Health Issue* (3.7%), consisted of adolescents who were likely to live with adults with mental/health issues. Last but not least, Class 4 was named *Multiple High Risk* (10.7%) class, comprising adolescents with a variety of ACEs with high likelihood, including a noticeably high rate of living with divorced or separated parents.

**TABLE 1:** Latent class analysis.

Indices		2‐Class model	3‐Class model	4‐Class model
BIC		86,429.88	82,104.73	81,777.85
Entropy		0.72	0.60	0.65
*p*‐values of LMR (VLMR)		< 0.001 (< 0.001)	0.13 (0.11)	0.69 (0.69)
5% rule		Satisfied	Satisfied	Not satisfied
Class ratio^a^	1	43.245%	28.387%	26.9%
	2	56.755%	12.725%	58.7%
	3	—	58.888%	3.7%
	4	—	—	10.7%

*Note*: Estimator: MLR (Maximum Likelihood Ratio).

^a^ Final class counts and proportions for the latent classes based on the estimated model.

### 4.2 RQ2: Differences in School Engagement Across ACE Class Memberships

Next, we investigated whether adolescents' levels of school engagement differed across four ACE class memberships, using a three‐step BCH approach in 4‐class model for latent class analysis [49]. An overall Chi‐square test was found to be a statistically detectable difference across four latent classes, *χ* ^2^ (3) = 137.74, *p* < 0.001. Specifically, follow‐up pairwise comparisons, adjusting significance level due to multiple comparisons (*α*_*adjusted*_ = 0.008) showed that adolescents assigned to the *No ACE* class had significantly higher school engagement scores than other three classes (i.e., *Multiple Low Risk*, *χ* ^2^ (1) = 17.61, *p* < 0.001; *Mental Health Issues* class, *χ* ^2^ (1) = 23.41, *p* < 0.001; *Multiple High Risk*, *χ* ^2^ (1) = 112.14, *p* < 0.001). Moreover, adolescents assigned to the *Multiple Low Risk* class showed significantly higher school engagement level than adolescents classified to the *Multiple High Risk*, *χ* ^2^ (1) = 48.48, *p* < 0.001. However, there were no significant differences in school engagement scores between the *Mental Health Issues* class and the *Multiple Low Risk* class (*p* = 0.01) and the *Multiple High Risk* class (*p* = 0.20). Descriptive statistics for school engagement across four ACEs groups are presented in Table 2.

**TABLE 2:** Descriptive statistics: School engagement by ACE class memberships.

ACE class memberships	*M* (SE)
1. No ACE	3.57 (0.04)
2. Multiple Low Risk	3.30 (0.03)
3. Mental Health Issues	3.00 (0.11)
4. Multiple High Risk	2.82 (0.06)

### 4.3 RQ3: Differences in School Engagement Across Intersecting Identities

Further, we investigated whether adolescents' levels of school engagement differed across the eight intersecting identities. A Wald Chi‐square test showed that school engagement differed across eight intersecting identities in a statistically detectable manner, *χ* ^2^ (7) = 200.68, *p* < 0.001. Follow‐up pairwise comparisons, adjusting significance level due to multiple comparisons (*α*_adjusted_ = 0.0018) showed that Asian female adolescents' school engagement was found to be the highest (*M* = 3.69, SE = 0.06), while White male adolescents' school engagement was the lowest (*M* = 3.13, SE = 0.02). White females were found to have significantly higher school engagement scores (*M* = 3.43, SE = 0.02), compared to the other three intersecting identity groups (i.e., Black males, *M* = 3.22, SE = 0.05; Hispanic males, *M* = 3.24, SE = 0.06; White males, *M* = 3.13, SE = 0.02). Table 3 includes descriptive statistics for school engagement across eight intersecting identities.

**TABLE 3:** Descriptive statistics: School engagement by intersecting identities.

Intersecting identities	*M* (SE)	Posthoc^a^
1. Asian‐Female	3.69 (0.06)	1 > 2, 4, 5, 6, 7, 8
2. Asian‐Male	3.50 (0.05)	2 > 4, 6, 8
3. Black‐Female	3.41 (0.08)	3 > 8
4. Black‐Male	3.22 (0.05)	4 < 7
5. Hispanic‐Female	3.40 (0.05)	5 > 8
6. Hispanic‐Male	3.24 (0.06)	6 < 7
7. White‐Female	3.43 (0.02)	7 > 8
8. White‐Male	3.13 (0.02)	—

^a^ The post hoc test results are shown in sequential order, with commas indicating pairs of groups that are found not to have a statistically detectable difference.

### 4.4 RQ4: Associations Between ACE Class Memberships and Intersecting Identities

To answer the fourth research question, a single Chi‐square test was conducted to examine whether each of the eight intersecting identities was associated with their ACE memberships^1^ [*χ* ^2^ (21) = 84.01, *p* < 0.001]. Specifically, Asian female adolescents and Asian male adolescents were more likely to be categorized into the *No ACE class*. Moreover, Black female and male adolescents were more likely to be classified into the *Multiple Low Risk* class and the *No ACEs* class than other classes. Likewise, a similar pattern was found in association with Hispanic female and male adolescents. While, overall, adolescents were less likely to live with parents with mental health issues, relatively higher rates of *Multiple High Risk* were identified in White and Black adolescents' households. See Table 4 for the frequency table between the ACE class memberships and intersecting identities.

**TABLE 4:** ACE class memberships by intersecting identities.

	Class 1^a^ (No ACE, 48.3%)	Class 2^a^ (Multiple Low Risk, 39.6%)	Class 3^a^ (Mental Health Issue, 2.6%)	Class 4^a^ (Multiple High Risk, 9.5%)
Asian‐Female (2.6%)	65.4% (3.5%)	30.8% (2.0%)	2.6% (2.6%)	1.2% (0.3%)
Asian‐Male (2.2%)	72.0% (3.3%)	26.0% (1.4%)	0.4% (0.3%)	1.6% (0.4%)
Black‐Female (7.3%)	31.9% (4.8%)	51.5% (9.5%)	2.5% (7.0%)	14.1% (10.8%)
Black‐Male (7.1%)	34.0% (5.0%)	55.1% (9.8%)	3.4% (9.3%)	7.5% (5.6%)
Hispanic‐Female (14.6%)	43.6% (13.2%)	44.3% (16.3%)	3.2% (17.7%)	9.0% (13.7%)
Hispanic‐Male (14.5%)	49.7% (15.0%)	40.8% (15.0%)	2.3% (13.1%)	7.1% (10.8%)
White‐Female (24.9%)	50.9% (26.2%)	35.5% (22.3%)	2.7% (25.8%)	10.9% (28.5%)
White‐Male (26.9%)	52.2% (29.0%)	34.9% (23.7%)	2.3% (24.2%)	10.6% (29.9%)

*Note*: The percentage indicates the proportion within the intersecting identities. The percentage in parentheses indicates the proportion within the ACE class memberships. *χ* ^2^ (21) = 84.01; *p* < 0.001.

^a^ ACE membership is based on their most likely latent class membership.

### 4.5 RQ5: Associations of ACE Class Memberships and Intersecting Identities With School Engagement

We conducted a hierarchical linear regression to test the associations between the ACEs memberships and their school engagement for each of the eight intersecting identities, controlling for socio‐demographics (e.g., poverty level, the highest education level of parents or caregivers in a household). Specifically, adolescents' socio‐demographics were controlled for in Step 1. In Step 2, adolescents' ACE class memberships were entered, with adolescents categorized into the *No ACE* class serving as the reference group. For each of the eight intersecting identities, in terms of Asian female, Hispanic male, White female, and White male adolescents, the full model was found to significantly predict school engagement, 0.06 ≤ *R* ^2^ ≤ 0.14. See Table 5 for significant standardized coefficients; significant coefficients after multiple comparisons correction (*α*_adjusted_ = 0.0021) are marked in Table 5. Table 6 includes descriptive statistics of school engagement across the combination of ACE class memberships and intersecting identities.

**TABLE 5:** Regression results (DV: School engagement).

	Asian‐Female		Asian‐Male		Black‐Female		Black‐Male		Hispanic‐Female		Hispanic‐Male		White‐Female		White‐Male	
	*B* (SE)	Beta	*B* (SE)	Beta	*B* (SE)	Beta	*B* (SE)	Beta	*B* (SE)	Beta	*B* (SE)	Beta	*B* (SE)	Beta	*B* (SE)	Beta
Step 1: Covariate only																
Poverty level	0.02 (0.05)	0.04	0.07 (0.06)	0.13	0.09 (0.05)	0.11	−0.06 (0.05)	−0.08	−0.14 (0.06)	−0.19*	0.08 (0.05)	0.11	0.06 (0.02)	0.09**	0.11 (0.02)	0.14***
Highest education	0.02 (0.04)	0.05	0.09 (0.06)	0.18	0.12 (0.11)	0.14	0.05 (0.07)	0.06	0.03 (0.06)	0.05	−0.04 (0.05)	−0.06	0.03 (0.03)	0.04	0.03 (0.03)	0.03
Model fit	*R* ^2^ = 0.01		*R* ^2^ = 0.08		*R* ^2^ = 0.05		*R* ^2^ = 0.01		*R* ^2^ = 0.03		*R* ^2^ = 0.01		*R* ^2^ = 0.01		*R* ^2^ = 0.02**	
Step 2: Covariate and ACE class memberships																
Poverty level	0.03 (0.04)	0.07	0.06 (0.06)	0.12	0.07 (0.05)	0.10	−0.06 (0.05)	−0.08	−0.13 (0.06)	−0.18	0.07 (0.05)	0.09	0.03 (0.02)	0.04	0.06 (0.02)	0.08**,^†^
Highest education	−0.01 (0.03)	−0.03	0.08 (0.05)	0.16	0.09 (0.07)	0.11	0.07 (0.07)	0.08	0.03 (0.06)	0.05	−0.02 (0.05)	−0.02	0.01 (0.03)	0.01	−0.01 (0.03)	−0.01
Class 1: No ACE^a^	—		—		—		—		—		—		—		—	
Class 2: Multiple Low Risk	−0.37 (0.11)	−0.33***,^†^	0.02 (0.13)	0.02	0.01 (0.11)	0.01	−0.08 (0.10)	−0.05	0.04 (0.10)	0.03	−0.30 (0.12)	−0.19**	−0.19 (0.04)	−0.13***,^†^	−0.29 (0.04)	−0.17***,^†^
Class 3: Mental Health Issues	−0.59 (0.37)	−0.18	−0.10 (0.39)	−0.01	−0.20 (−0.74)	−0.04	−0.48 (0.25)	−0.11	−0.12 (0.27)	−0.03	−0.42 (0.26)	−0.08	−0.54 (0.12)	−0.13***,^†^	−0.46 (0.12)	−0.09***,^†^
Class 4: Multiple High Risk	−0.39 (0.14)	−0.09*	−0.83 (0.47)	−0.17	−0.74 (0.28)	−0.32**	−0.52 (0.18)	−0.18**	−0.20 (0.16)	−0.08	−0.57 (0.11)	−0.19***,^†^	−0.45 (0.07)	−0.20***,^†^	−0.74 (0.06)	−0.29***,^†^
Model fit	*R* ^2^ = 0.14*		*R* ^2^ = 0.11		*R* ^2^ = 0.16		*R* ^2^ = 0.04		*R* ^2^ = 0.04		*R* ^2^ = 0.07*		*R* ^2^ = 0.06***		*R* ^2^ = 0.11***	

^a^ Reference group.

* *p* < 0.05.

** *p* < 0.01.

*** *p* < 0.001.

^†^ Significant coefficient when adjusted alpha applied.

**TABLE 6:** Descriptive statistics of school engagement (*M*, SD) [*n* = sample size].

	Asian‐ Female	Asian‐ Male	Black‐ Female	Black‐ Male	Hispanic‐ Female	Hispanic‐ Male	White‐ Female	White‐ Male
Class 1: No ACE	3.82 (0.04) [*n* = 280]	3.53 (0.07) [*n* = 311]	3.53 (0.09) [*n* = 184]	3.32 (0.07) [*n* = 191]	3.39 (0.08) [*n* = 451]	3.42 (0.08) [*n* = 511]	3.57 (0.03) [*n* = 2481]	3.33 (0.02) [*n* = 2746]
Class 2: Multiple Low Risk	3.45 (0.11) [*n* = 134]	3.48 (0.09) [*n* = 127]	3.54 (0.06) [*n* = 296]	3.25 (0.08) [*n* = 315]	3.47 (0.08) [*n* = 432]	3.11 (0.09) [*n* = 508]	3.37 (0.03) [*n* = 1711]	3.02 (0.04) [*n* = 1949]
Class 3: Mental Health Issues	3.23 (0.37) [*n* = 15]	3.44 (0.32) [*n* = 4]	3.37 (0.17) [*n* = 25]	2.84 (0.24) [*n* = 26]	3.29 (0.24) [*n* = 42]	2.95 (0.23) [*n* = 41]	3.02 (3.11) [*n* = 161]	2.86 (0.12) [*n* = 141]
Class 4: Multiple High Risk	3.43 (0.15) [*n* = 8]	2.44 (0.45) [*n* = 8]	2.73 (0.32) [*n* = 62]	2.79 (0.16) [*n* = 57]	3.19 (0.14) [*n* = 114]	2.86 (0.09) [*n* = 137]	3.09 (0.06) [*n* = 553]	2.55 (0.05) [*n* = 570]

## 5 Discussion

Using a large dataset from the 2021 National Survey of Children's Health (NSCH), we aimed to investigate the role of ACEs and intersecting identities on school engagement among adolescents aged 12–17 in the United States. Despite some limitations associated with analyzing secondary data (e.g., lack of control over specific survey items), our results offered nuanced insights into how adolescents' contextual (i.e., ACEs) and individual (i.e., intersecting identities) factors independently and interactively influenced school engagement.

### 5.1 ACE Class Memberships and Their Associations With Intersecting Identities

We identified four distinct classes of ACEs: *Multiple High Risk*, *Multiple Low Risk*, *Mental Health Issues*, and *No ACE* classes, with the *No ACE* class serving as the reference group (RQ1). We further examined the associations between ACE class meberships and intersecting identities (RQ4). Each class had varying proportions of adolescents based on their intersecting identities. Specifically, the highest proportion across all intersecting identity groups (except Black males and females) was in the *No ACE* class, with Asian female and male adolescents having the largest proportion. Beyond the *No ACE* class, Black and Hispanic adolescents, both male and female, had the largest proportion in the *Multiple Low Risk* and *Multiple High Risk* classes compared to other intersecting identity groups. These results align with previous studies indicating that Black and Hispanic adolescents are more likely to experience a wider range of adversities, compared to other racial/ethnic groups [37, 38]. In the *Mental Health Issues* class, Black male and Hispanic female adolescents showed the largest proportion, consistent with earlier research [50, 51].

### 5.2 Differences in School Engagement Across ACE Class Memberships and Intersecting Identities

We found significant differences in the levels of school engagement across the ACE class memberships (RQ2) and intersecting identities (RQ3) separately. Regarding the ACE class memberships, adolescents in the *No ACE* class had the highest levels of school engagement, while those in the *Multiple High Risk* class were found to have the lowest levels. This is not surprising in that these results are aligned with the dose–response relationship between the number of ACEs and increased risks of disengagement in school [12, 23]. Within intersecting identities, Asian female adolescents showed the highest levels of school engagement, while White male adolescents demonstrated the lowest levels of school engagement. Indeed, prior work has shown that Asian female adolescents demonstrated academic success and adherence to educational values, attributed to both gender and cultural factors [13, 52]. However, the results of White male adolescents were somewhat unexpected. One possible explanation is that White male adolescents comprised the largest proportion of the sample, which may have amplified within‐group variation and influenced the overall trend. Additionally, factors, such as other sociodemographic backgrounds and environmental contexts, could further shape these engagement patterns [9, 53, 54].

### 5.3 Role of ACEs Class Memberships and Intersecting Identities on School Engagement

Our results highlighted the combined impacts of ACE class memberships and intersecting identities on adolescents' school engagement, which only partially aligned with the results when ACEs and intersecting identities were examined separately. Specifically, when both ACE memberships and intersecting identities were considered, Asian male, Black female, Black male, Hispanic male, and White male adolescents in the *Multiple High Risk* class demonstrated lower levels of school engagement when compared across all intersecting identity groups. These results were consistent with the earlier analysis that examined ACEs alone, where the *Multiple High Risk* class also showed the lowest school engagement. The alignment across the two analyses also supported the dose–response relationship between the number of ACEs and the increased risk of school disengagement [12, 23]. However, the differences emerged in terms of intersecting identities. When ACEs were excluded and intersecting identities were examined alone, White male adolescents reported the lowest levels of school engagement. When both ACEs and intersecting identities were included, Asian male adolescents showed the lowest engagement levels, despite their small sample size, followed by White male adolescents. Further, Black female adolescents, who had the third highest engagement when only intersecting identities were considered, had the third lowest when ACEs were included. These different results emphasize how the effects of intersecting identities on school engagement differ depending on the inclusion of contextual factors, such as ACEs. These contrasting results emphasize the importance of examining both ACEs and intersecting identities together to better understand patterns of school engagement among adolescents from various backgrounds.

While our results provide a foundation for more holistic research and follow‐up interventions that consider contextual and individual factors in explaining adolescents' school engagement, future research should explore these influences more deeply. Prior research has shown that school engagement tends to decline from middle to high school [10, 11], with older adolescents generally exhibiting lower levels of engagement. This decline may stem from the challenges of transitioning into more advanced academic levels [55] and differences in school climates [56]. Since our study included adolescents aged 12–17, spanning both middle and high school, future studies should investigate whether engagement patterns differ between middle school and high school students. Further, the school environment may play a role in shaping students' levels of school engagement. For instance, school size has been shown to impact students' engagement, as students in moderately sized high schools benefited from higher engagement levels. In contrast, cohorts exceeding 400 students were associated with potential negative effects on engagement [57]. Since our study provided a broad analysis without accounting for developmental and environmental (e.g., community or school) differences, future research should investigate these factors to provide a more nuanced understanding of how these factors contribute to school engagement for each student group.

Together, these insights can enhance educational interventions, such as trauma‐informed teaching practices and social–emotional learning programs [58, 59, 60]. For instance, culturally responsive services have been criticized for oversimplifying cultural celebrations or essentializing a group's traditions or cultures [61]. To address these limitations, applying a more holistic framework that considers both ACEs and intersecting identities can offer deeper insights into students' school engagement. Similarly, prior work introduced a culturally‐informed ACEs (C‐ACEs) framework that incorporates both contextual (e.g., ACEs) and individual (e.g., race) factors to explain mental health issues among culturally marginalized youth [62]. While our study focused on ACEs and intersecting identities, future research could explore how additional factors, such as developmental stages and school environmental contexts, further shape school engagement. Expanding these frameworks in educational settings could foster more supportive learning environments.

### 5.4 Implications for School Health Policy, Practice, and Equity

Our results highlight the importance of integrating intersecting identities [27] and ecological perspectives [14] into school policies and practices to promote school engagement. Specifically, the significantly lower levels of school engagement among Black female adolescents in the *Multiple High Risk* group in our study suggested the need for systemic supports with culturally responsive and evidence‐based approaches. At the policy level, schools should institutionalize support for culturally responsive and trauma‐informed programs, such as “Sisters of Nia” [63, 64], designed to build ethnic identity, promote school engagement, and build resilience. Such policy support can help ensure these programs are consistently implemented and accessible.

Similarly, policies that require regular training for teachers and staff on ACEs and trauma‐informed interventions can also enhance school‐wide capacity to support diverse students [65, 66]. Such training is recommended to be embedded into school calendars and to include content on the neurological impacts of ACEs, practical intervention strategies, and self‐care practices [67, 68]. Further, school policies should support teacher wellness by providing mental health days, access to peer support, and stress‐reduction programs (e.g., mindfulness or yoga) [68, 69]. These policies help educators sustain the emotional energy needed to support marginalized and traumatized students.

In terms of school health practice, school health professionals can implement targeted psychoeducational support groups to enhance resilience and school engagement among adolescents experiencing parental substance use. School‐based support groups providing education, peer support, and safe environments for sharing have been shown to effectively increase resilience in affected adolescents [70]. Additionally, structured psychoeducational interventions addressing parental opioid use have improved adolescents' emotional regulation, behavioral skills, and academic engagement [71]. By incorporating these interventions into school's regular practice, educators can create stable and responsive environments that meet the needs of students facing adversity.

Lastly, to ensure that these efforts remain effective, schools should adopt data‐driven approaches that respond to the diverse and evolving needs of their students. Regular needs assessments and evaluations can guide the continuous improvement of trauma‐informed and culturally responsive policies and practices, especially for students impacted by structural (e.g., poverty, limited access to mental health care) and familial adversity [72]. By aligning school health policies and practices with support for ACEs‐ and trauma‐related resources—using culturally responsive and evidence‐based approaches—school teachers and staff can enhance tailored interventions to improve school engagement among traumatized students, ultimately fostering more supportive school environments.

## 6 Conclusion

In conclusion, this study highlights the distinct yet interconnected impacts of contextual factors (e.g., ACEs) and individual factors (e.g., intersecting identities) on adolescents' school engagement. To bridge gaps between students from various backgrounds and foster more supportive educational environments, we suggest follow‐up studies focusing on the specific subgroups identified in this study. Such research should guide the enhancement of tailored interventions, such as culturally responsive services, trauma‐informed teaching practices, and social–emotional learning programs. Ultimately, these targeted, holistic approaches are crucial for fostering a supportive educational environment that promotes academic success for all adolescents in the United States.

## Ethics Statement

We did not seek IRB approval because this study used secondary data collected by the NSCH, which already directly obtained ethical approval and informed consent from study respondents.

## Conflicts of Interest

The authors declare no conflicts of interest.

## Data Availability

The data that support the findings of this study are openly available in NSCH data at https://www.childhealthdata.org/learn‐about‐the‐nsch/NSCH/data.

## Endnotes

^1^
For RQ1–RQ3, ACE membership is based on the estimated model. For RQ4 and RQ5, ACE membership is based on their most likely latent class membership.
